# Influence of Opium Habit on Menstruation

**Published:** 1889-09

**Authors:** B. M. Wooley

**Affiliations:** Ga.


### INFLUENCE OF OPIUM HABIT ON MENSTRUATION.

By B. M. Wooley, M. D., of Ga.

Noting the article of Dr. Eutaud in *N. Y. Med. Record* of July 22d, I would like to say that I have had extensive observation in regard to the action of morphine in the particular here referred to, and can confirm, to a large extent, what he says about it, only making this correction, that I do not agreee with him that it entirely destroys the functions. It does check ovulation and suppress menstruation, but I find that, on treating them for the morphine habit, in which I have had the observation in my practice of over a thousand cases, the sexual desire is not only returned, but a curious fact is that menstruation returns promptly. To illustrate. In cases of ladies thirty and thirty-five years of age, regularly menstruating when they get into the opium habit, in a short time menstruation is suppressed and the sexual desire, to a large extent extinguished, but when these cases have reached fifty and even fifty-five years with the menstruation still suppressed under the influence of the opiates, when the opiates are removed and the system properly treated for the damages sustained by opium, the menstruation returns and seems as normal as when the habit was first contracted. And, in the majority of cases, they will conceive and have children. I have had many such cases. This would seem that it does not destroy but simply suspends the function.

As to his suggestion, that morphine would prove of service in the treatment of women suffering from affections aggravated by the menstrual flow, such as fibroid, &c., no doubt he is correct. Of course, it will generally result in the patient's getting into the morphine habit. I will say this, that if he will combine the fluid extract of black haw (viburnum prunifolium) and the fluid extract of red shank (ceanothus Americanus), in equal quantities and then give in drachm doses, every two or three hours, he will find a most useful adjunct to the use of opiates in such cases, especially where there are profuse uterine hemorrhages.

